# A COVID-19 Lockdown Tabletop Exercise in New Taipei City, Taiwan

**DOI:** 10.1017/dmp.2021.35

**Published:** 2021-02-16

**Authors:** I-Tien Lo, Ching-Yuan Lin, Ming-Tai Cheng

**Affiliations:** 1 Department of Architecture, National Taiwan University of Science and Technology, Taipei, Taiwan; 2 Disaster Management Office, New Taipei City Government, New Taipei City, Taiwan; 3 Department of Emergency Medicine, National Taiwan University Hospital, Taipei, Taiwan; 4 Taipei Regional Emergency Medical Operation Center, Ministry of Health and Welfare, Taipei, Taiwan

**Keywords:** COVID-19, lockdown, public health, tabletop exercise

## Abstract

**Objectives:**

This exercise aimed to validate New Taipei City’s strategic plan for a city lockdown in response to coronavirus disease (COVID-19). The main goal of all solutions was the principle of “reducing citizen activity and strengthening government control.”

**Methods:**

We created a suitable exercise, creating 15 hypothetical situations for 3 stages. All participating units designed and proposed policy plans and execution protocols according to each situation.

**Results:**

In the course of the exercise, many existing policies and execution protocols were validated. These addressed (1) situations occurring in Stage 1, when the epidemic was spreading to the point of lockdown preparations; (2) approaches to curb the continued spread of the epidemic in Stage 2; and (3) returning to work after the epidemic was controlled and lockdown lifted in Stage 3. Twenty response units participated in the exercise. Although favorable outcomes were obtained, the evaluators provided comments suggesting further improvements.

**Conclusions:**

Our exercise demonstrated a successful example to help policy-making and revision in a large city of over 4 million people during the COVID-19 pandemic. It also enhanced participants’ subject knowledge and familiarity with the implementation of a city lockdown. For locations intending to go into lockdown, similar tabletop exercises are an effective verification option.

## Introduction

On January 21, 2020, the Central Epidemic Command Center (CECC) announced the first confirmed imported case of novel coronavirus infection (coronavirus disease; COVID-19) in Taiwan,^1^ and the first confirmed, locally acquired case was reported on January 28.^2^ Since then, Taiwan has actively promoted and adopted the concept of epidemic prevention. This includes advocating the frequent washing of hands, wearing of surgical masks, and avoiding crowded places; additionally, incoming travelers are required to observe home quarantine for 14 days. The epidemic situation remained stable and under efficient control until April 17; on the following day, a cluster infection of COVID-19 was reported within the Navy crew aboard the fast combat support warship, “Pan-Shi,” which rendered the epidemic situation in Taiwan unstable again. At this point, COVID-19 was proliferating in Italy, Spain, the United States, and worldwide. On April 27, the cumulative confirmed cases numbered more than 3 million.^3^ Many countries and municipalities started to implement different levels of lockdown guidance measures.

From the perspective of public health preparedness, however, uncertainty about the future development of the epidemic and its further spread remained. To prepare for the possibility of a pandemic in the future, the New Taipei City Government created a plan for city lockdown in response to the COVID-19 pandemic over the course of 2 weeks. The plan was developed by 7 major administrative departments and was intended for execution by 28 departments, 29 district offices, and the military. The practical feasibility of the plan remains unclear.

The state of Maryland (United States) held a tabletop exercise in April 2004 to test its pandemic preparedness plans and identify any remaining gaps in its plans.^4^ The state of Massachusetts (United States) carried out a similar exercise.^5^ Two adjacent counties in the state of Arkansas (United States) held a joint tabletop exercise to simulate the public health response to a severe acute respiratory syndrome (SARS) event.^6^ Such tabletop exercises provide opportunities to identify and solve existing problems,^7^ test contingency plans, and review the decision-making process. They can also test the basic capabilities of contingency units and their teamwork^8^ and have proven to be a suitable and feasible low-cost approach in disaster medicine.^9^

Tabletop exercises can take many different forms and may consist of several modules,^10^ sample questions,^8^ or scripted scenarios^6^; for example, in the Maryland exercise, participants were presented with 9 different fictitious scripts encompassing a single scenario.^4^ Participation may be open to public health workers, government officials, private organizations, senior professionals with decision-making power, and senior leaders.^4,8,10^

COVID-19 has proven to be a highly contagious disease with serious medical complications and, to date, continues its worldwide pandemic spread,^11^ similar to the previous influenza and SARS events. To validate the feasibility of New Taipei City’s COVID-19 lockdown plan, the city government thus decided to hold a tabletop exercise.

## Tabletop Exercise Planning Process

Any tabletop exercise scenario must go through a design and discussion phase. The design is based on the problems that may be faced, such as disseminating information about the epidemic situation during the lockdown, supplying daily necessities to civilians, restricting the activities of personnel, halting work and school attendance, and closing public places and nonessential businesses. The specific objectives and the required scale dictate the actual design. The main goal is to identify possible problems, test the feasibility of the lockdown policy, and assess the ability of the response unit to deal with arising contingencies. The steps in the following sections were carried out in planning the present exercise.

### Step 1. Scenario Assumptions, Goals, and Scope

The results of the lockdown measures implemented in Wuhan, China, show that the implementation of stringent restrictions effectively slowed the spread of COVID-19.^12^ How well a community fares under such circumstances depends on how early lockdown measures are introduced.^13^ Therefore, this tabletop exercise aimed to derive solutions to quickly stop the spread of COVID-19 in New Taipei City after an outbreak. Scenario assumptions for the exercise were formulated based on the data provided by the Public Health Department, New Taipei City Government. In New Taipei City, lockdown preparations are set to be initiated when the basic reproduction number (R0) of COVID-19 reaches 1.5.^14^ In this situation, all response units undergo lockdown preparations, take inventory of their resources, and formulate control policies and countermeasures. Lockdown measures are implemented immediately once R0 = 2.0.

### Step 2. Scenario Design

Lockdown measures are based on regional laws, cultures, geographical features, lifestyles, and economic models. They must be adapted to local conditions. Some researchers have recommended applying Maslow’s hierarchy of needs to this analysis.^15^ Based on the laws, cultures, lifestyles, and economic models of Taiwan, the tabletop exercise focused on satisfying people’s basic needs after the implementation of lockdown measures, taking into account the following 6 dimensions in the formulation of plans and policies:

1. Food: How will people obtain food and daily necessities?
2. Health: How will people receive medical assistance when they fall ill?
3. Accommodation: Are people’s homes safe to carry out stay-at-home orders, and are there sufficient quarantine hotels?
4. Transportation: How will people get around?
5. Education: How will students continue their education after school closures?
6. Entertainment: Will entertainment be provided after lockdown measures are implemented?

All scenarios and situations were designed to “reduce people’s activity” and “strengthen government regulations.” Therefore, we derived 6 main measures and policies that should be established:

1. Essential resources: Supply and acquisition of food and daily necessities
2. Commerce: Continuation of essential businesses and critical infrastructure
3. Medical care and rescue: Continuation of the health care system, environmental sanitation and disinfection, and emergency rescues
4. Personnel management: Classification of essential workers from among the general public
5. Traffic control: Operation of mass transportation and establishment of road traffic control points and modes of transportation
6. Education and media: Continuation of education and distribution of information concerning measures and policies

### Step 3. Response Unit Preparation

Once the scenario assumptions and situation simulations characterized in Step 2 are set, the participating response units take an inventory of their resources and then formulate control policies and execution protocols based on the simulated situations. If a response unit lacks the resources to resolve a situation or is incapable of resolving the situation, it must design and implement an alternative solution and review the feasibility of its control policies and execution protocols. If an execution policy produces unfavorable outcomes, it should be avoided or resolved.

### Step 4. Rehearsal and Feedback

To ensure that the scenarios of the tabletop exercise conform to real-world situations and that the policies, plans, and execution protocols formulated by the response units coincide with the scenarios, participants were required to rehearse the tabletop exercise, provide feedback, and engage in discussions before engaging in the formal tabletop exercise. Scenario assumptions were revised during each rehearsal to maximize the conformity between the assumptions and real-world situations. Corrections must be made when feedback fails to coincide with real-world situations. All scenario assumptions and policy formulations must conform to the primary scenario design concept.

## Methods

For the present exercise, we designed 15 situations within the 3 stages and formulated the corresponding response units (Table 1). The 3 stages were set based on the chronological development of epidemics and relevant lockdown strategies. In the first stage, the epidemic has spread to the point of preparing for lockdown. In the second stage, the epidemic has continued to spread to the point of an actual lockdown. In this stage, approaches to curb the continued spread of the epidemic and satisfy the basic needs of the citizens during lockdown are devised. In the third stage, the spread of the epidemic has been controlled. In this stage, strategies for safely returning to work after lockdown are devised.

**Table 1.** Situation and emergency response units for the tabletop exercise

Stage	Situation	Emergency Response Units
Stage 1 before lockdown	Epidemic intelligence and preparedness for lockdown	B
	Establishing an emergency operation center	A, C
	Preparedness for daily necessities	D, G, E, J, F, P, B, K, O, L, I
	Epidemic intelligence and lockdown announcement	B, T, D, E, F
	Strategy for panic buying	E, G
Stage 2 during lockdown	Personnel control and imposing restrictions	D, F, G, H
	Transportation control and measures to restrict traffic	F, P, G, I
	Inspection of essential businesses	E, J, G
	Medical care services and operation of the health system	B, K, F, L
	Educational disruption and response	M
	Care for underprivileged groups	N
	Environmental cleaning and waste disposal	O
Stage 3 deregulation	Epidemic intelligence and deregulation	B
	Environmental cleaning and disinfection	O, I
	Recovery and financial relief	M, F, P, E, Q, R, S

*Notes*: Emergency response unit codes —A: Disaster Management Office, B: Department of Health, C: Banqiao District Office, D: Department of Civil Affairs, E: Department of Economics Development, F: Department of Transportation, G: Department of Police, H: Zhonghe District Office, I: National Military, J: Department of Agriculture, K: Fire Department, L: Department of Travel, M: Department of Education, N: Department of Social Welfare, O: Department of Environmental Protection, P: Department of Rapid Transit Systems, Q: Department of Labor Affairs, R: Department of Budget, Accounting and Statistics, S: Department of Information, T: Department of Personnel.

## Selecting the Tabletop Exercise

A suitable exercise must first be selected to accurately verify the feasibility of the COVID-19 lockdown strategy for New Taipei City and highlight potential problems. Not only would an inappropriate exercise fail to truly verify the lockdown strategy, but also it would be unable to cover and achieve the specific goals and focuses. Therefore, we selected a suitable exercise based on (1) suitability of the approach to fulfill the objective, (2) length of time required to carry out the exercise, (3) size of the selected venue, (4) number of participants, and (5) expected results. Exercise participation time should be minimized during a pandemic.

Therefore, the execution time of this exercise was limited. To adhere to pandemic requirements, we originally planned to pitch outdoor tents in the exercise venue. However, the additional equipment and preparation time required would be overly cost- and time-intensive. As an alternative, we found a venue with a capacity for 100 people. We ensured that the venue was well-ventilated and that participants adhered to social distancing protocols. Due to the time and participant constraints, the New Taipei City Government designated response units based on pre-selected situations and problems, and appointed decision-makers from these units to participate in the exercise. These response units formulated strategies to address potential problems that could arise after lockdown.

## Setting Appropriate Scenarios and Seeking Expertise

The correct and appropriate scenarios are crucial for fulfilling the objectives of this exercise. Scenario design is discussed in Step 2 of the tabletop exercise planning process. In addition to setting exercise objectives and proposing 6 major measures and policies, we continued collecting information concerning the development of the COVID-19 pandemic throughout the design process and reviewed the control measures and policies concerning COVID-19 in various countries. We then reviewed relevant laws, regulations, and literature to revise the scenarios and devise various situations. Finally, we identified the ideal policy and solution through rehearsal and feedback collection, as characterized in Step 4.

Expertise was the basis for designing scenarios and proposing solutions, particularly those involving epidemic features and transmission methods. Effective plans are only possible when they are created with expert knowledge. Therefore, the scenarios of this exercise were designed across multiple meetings, drawing from and consolidating the knowledge of the expert representatives from the 7 major planning and response units.

## Execution of the Tabletop Exercise

**Figure 1.**
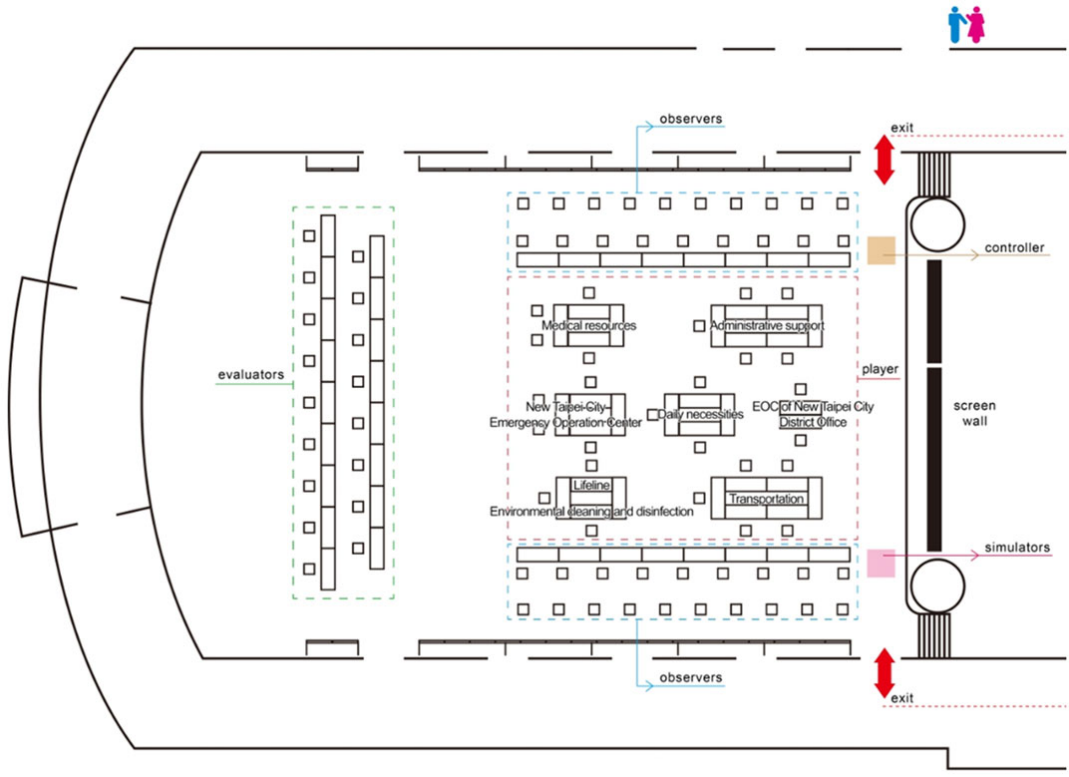
Layout of exercise venue.

Participants rehearsed the tabletop exercise based on the assumptions and situations. The tabletop exercise comprised 3 stages and 15 scenarios. Twenty response units participated in the exercise: 1 city office, 16 departments, 2 district offices, and a military unit in New Taipei City. The location of the simulation was New Taipei City, which covers a land area of 2052 km^2^, and the duration of the simulation was 21 days. The layout of the exercise venue is illustrated in Figure 1.

COVID-19 is primarily spread from person to person at close range. Therefore, people are the main targets of control when implementing lockdown measures. These control measures categorized people into “essential workers” and “general public.” Essential workers are permitted to go outside for work (Table 2). These workers are authenticated by their employee identification card and their National Identification Card. Essential workers without employee identification cards are issued a permit by the transportation control authorities. An identification system will be implemented to prevent counterfeiting. The general public is permitted to go outside only during designated periods to purchase food and daily necessities (Table 3). The designated period for each group of people is based on the last digit of their National Identification Card. Each person is permitted to go outside 3 times a week. Children under the age of 13 are required to stay indoors.

**Table 2.** Essential workers in critical infrastructure sectors

Essential Workers in Critical Infrastructure Sectors
1. Workers in government and affiliated offices
2. Workers in medical care, public health, and veterinary medicine
3. Workers in food production and supply
4. Workers in critical infrastructure operations
5. Workers in transportation and logistics
6. Essential workers operating public infrastructure and facilities
7. Essential workers in communication and information technology
8. Essential workers in property management and security

**Table 3.** Schedule for when members of the general public are allowed to leave their homes

Weekday	Monday	Tuesday	Wednesday	Thursday	Friday	Saturday	Sunday
Time of day	Last number of ID card						
8:00 AM to 1:00 PM	1, 2	5, 6	9, 0	3, 4	7, 8	1, 2, 3	6, 7
2:00 PM to 7:00 PM	3, 4	7, 8	1, 2	5, 6	9, 0	4, 5	8, 9, 0

*Notes*: 1. Excursion is only allowed for buying household products and food to be consumed at home. 2. Any person over 13 years old can go out, but person cannot accompany other people and must carry an ID card.

The tabletop exercise was carried out on April 20 and lasted for 70 minutes. Government officials from the CECC and neighboring cities were invited to share their experiences. The event was limited to 100 attendees to meet social distancing and venue capacity restrictions, although these restrictions affected the number of players, simulators, observers, and evaluators.

## Results

All 20 response units were able to provide feedback based on the scenario assumptions and situations during the 70-minute tabletop exercise. Although favorable outcomes were obtained, the experts and evaluators provided the following comments:

1. First, citizen activity policies must be flexible. The policies and execution protocols are established to restrict people’s activity. However, these policies and protocols should be adjusted as the conditions of the epidemic change over time. These adjustments were not presented or explained in this tabletop exercise.
2. Second, the central government should collaborate with local governments to implement the health system and formulate a comprehensive plan for the operation of the health system.
3. Finally, lifting restrictions should be a gradual process rather than a 1-time deregulation.

In addition to the opinions of the evaluators, we found that the participants were unable to fully respond to or explain many of the scenario assumptions due to the time constraints of the tabletop exercise. Our observations coincided with the views of the evaluators. Although the participants provided feedback concerning the main policies and execution protocols for each scenario and verified the feasibility and flaws of the lockdown plan, they failed to put forward flexible execution approaches. Furthermore, participants were pre-selected to respond to specific scenario assumptions. However, several divergent situations were not part of the initial design. Thus, the controller raised ad hoc questions and selected participants to respond to these questions.

## What Went Well

The tabletop exercise showcased many peri-epidemic policies and execution protocols, including lockdown initiation standards, essential resource supply and preparation, public activity restrictions, school and business closures, continuation of compulsory education, medical system operation, closure of public spaces, and suspension of non-essential business activities, as well as post-epidemic policies, such as opening public spaces, recommencing business activities, and economic relief plans. The exercise provided the opportunity to comprehensively simulate the implementation of lockdown measures amidst the COVID-19 epidemic from pre-lockdown preparations to post-lockdown deregulation. Overall, the tabletop exercise was a success. It achieved the objective of validating the feasibility and identifying the flaws of lockdown plans. Although certain aspects of the exercise require further deliberation, a consensus was achieved among the participants. Through promotions of the CECC and the various government departments and offices and dissemination through news outlets, the residents of New Taipei City can gain a preliminary understanding of the city’s lockdown plan.

## Lessons Learned

In this exercise, we also found that, although response units adopted the same policies, the details of the execution protocols were inconsistent. Many arrangements, such as the planning of traffic control posts and shift schedules, were different between the police department and district offices, which caused confusion over the traffic control policy because each department had their own rules. After this exercise, each execution protocol in New Taipei City was requested to be unified by a single responsible department of the municipal government. For example, in accordance with the conclusion of the exercise, the control policy for crowds in tourist attractions was assigned to the “Travel Department” of New Taipei City by the mayor, and other relevant units, such as the police, economic development, and district offices, will now need to cooperate with the Travel Department to avoid confusion. Moreover, New Taipei City began to close certain indoor public places and has restricted various social activities since March 20. Owing to the comments of infectious disease experts and other specialists in this exercise, the lifting of the restriction protocols was revised to be a gradual process consisting of 2 stages and 4 batches, rather than a 1-time deregulation, as it had been since May 4. In the first stage of lifting restrictions, only activities and places that could well-implement health monitoring, personnel management, flow control, good ventilation, and the maintenance of social distancing were included. The first batch of Stage 1 lifting restrictions was only for individuals, and the second batch was for group activities with less than 10 people. In the second stage, the lifting restrictions included all activities and places. The lifting restrictions in the first batch of Stage 2 were for indoor activities for groups less than 100 people without sharing food, and outdoor activities for groups less than 500 people. The second batch was full deregulation, such as school graduation ceremonies and relaxation of the flow control at tourist attractions. Additionally, the implementation of the above protocols should also contain the maintenance of social distancing, advocation for the frequent washing of hands and wearing of face masks, and avoidance of overcrowded places. The COVID-19 lockdown strategy of the New Taipei City Government was partially adjusted after the tabletop exercise, as shown in Table 4. A number of changes have already been implemented.

**Table 4.** Policy revisions after the tabletop exercise

Item	Before the Exercise	After the Exercise	Status
Traffic control policy	Independently designed by relevant units	Designed by the police department with the help of the other units	Lockdown has yet to be instated
Crowd control at tourist attractions	Independently designed by relevant units	Designed by the tourism department with the help of the other units	Implemented
Lifting restrictions	One-time lift	Gradual lift	Implemented

## Discussion

Tabletop exercises have been carried out to raise awareness about public health emergencies and help government officials and relevant actors understand the contingencies.^5^ The described tabletop exercise is the first such exercise carried out in Taiwan for COVID-19. It involved legal, transportation, business, health, and education policies. COVID-19 has not developed into a pandemic in Taiwan, and potential situations stemming from a pandemic situation are therefore difficult to validate. In 2003, Taiwan was severely affected by the SARS outbreak. We designed several hypothetical scenarios to be close to real-world situations, based on the local experiences with SARS and on the data on COVID-19 shared by various countries. These scenarios were tested by a number of response units. Due to time constraints, we were able to simulate only primary policies and their execution protocols. The preliminary exercise objectives were achieved. However, the execution protocols should be reconceived and redesigned to include greater detail.

There were several limitations of this exercise. First, the formal exercise time was short. Due to the time constraints, the participants were given sufficient time beforehand to discuss the scenarios and feasible solutions and achieve a preliminary consensus. These solutions were then tested during the formal exercise. The participants of the exercise as players were chiefs or decision-makers in their departments, and thus they were the most aware of the resources and capabilities of their departments. The scenarios were as realistic as possible; participants were therefore able to achieve the objectives of the exercise and then to realize and modify their policies and protocols through multi-unit interactions on the scene. Although the policies were still not perfect in this exercise, they were acceptable after this coordinated effort.

Second, the response units of nearby cities and the central government did not participate in the exercise as players, except for military units. The impact of lockdown is not limited to New Taipei City but will also be felt by nearby cities. After the municipal response units formulate policies and execution protocols, the collaboration of nearby cities and the central government is crucial. Here, we invited representatives of nearby cities to participate as observers and experts from the central government to participate as evaluators and give comments. In addition to hearing their opinions, we had also hoped to promote a mutual understanding and create a basis for future collaborations.

Various units of the municipal government also cultivated tacit understandings during the exercise. In addition to understanding the main duties of other response units, they can realize their own responsibilities more clearly, and cooperation will be closer. In our exercise, the execution protocols of 1 policy were formally unified by the main responsible department through the discussion, which improved the clarity and efficiency of the division of work. Such achievements are very worthwhile. Each response unit of the municipal government does not need to actually implement the various protocols, nor do the response units need to use much labor power or resources to obtain the desired results.

The objectives of lockdown are to strengthen government control and reduce public activity, with the aim of immediately stopping the spread and outbreak of COVID-19 and avoiding an overload of the health system while minimizing the effects of the epidemic on people’s daily lives. An epidemic of this type cannot be controlled without effective measures. The outbreak of an epidemic could cause immeasurable adverse consequences, and restricting people’s outdoor activities and minimizing social interactions are crucial for stopping its spread. The concept of the proposed exercise is similar to the lockdown instated in Wuhan. Other countries chose to implement more relaxed restriction policies. The outcomes of Wuhan’s lockdown show that more stringent policies to limit people’s outdoor activities are more effective at controlling the development of epidemics.^12^

Fortunately, the COVID-19 epidemic in Taiwan has not required a true lockdown situation so far. Therefore, through this city-government level tabletop exercise, we also achieved good policy discussions and interactions among many departments at short notice. In addition, tabletop exercises can be carried out systematically and can be adapted for use by other departments.^6^ The outcomes, scenario designs, and response protocols from these exercises have significant reference value for other cities in Taiwan. Accordingly, tabletop exercises were also carried out in Taichung City and Taipei City on April 24 and April 30, 2020.

## Conclusion

Against a background of global attempts to devise control measures for and responses to COVID-19, the proposed tabletop exercise is an effective approach for various government departments to examine the execution of lockdown measures in detail. Our exercise demonstrated a successful example to help policy-making and revision in a large city of over 4 million people during the COVID-19 pandemic. It also enhanced participants’ subject knowledge and familiarity with the implementation of a city lockdown. The findings of this study indicated that rehearsal and feedback collection before the formal exercise maximized the conformity between the assumptions and real-world situations. Therefore, the outcomes of the exercise were extremely beneficial. Moreover, selecting a suitable exercise is equally important. In this study, we directly established the scenarios and problems, and the decision-makers of the designated response units were appointed to participate in the exercise. These settings enabled us to determine the expected results without the need to undergo complicated administrative procedures to confirm the abilities of the decision-makers and the direction of the policies.

The experience gained and lessons learned from the exercise are as follows. First, each response unit planned independently for its assigned tasks. However, for joint tasks (eg, traffic control), a lead unit will be selected to plan the task and will be supported by other units. This process eliminates confusion. Second, the central government must collaborate with local county and city governments when implementing lockdown protocols to ensure effectiveness. Finally, the re-opening process should be carried out gradually rather than as a 1-time lift of all restrictions to prevent the resurgence of the epidemic.

Tabletop exercises provide a low-cost solution for discussing contingencies in countries facing imminent lockdown or that are already in lockdown. Such exercises also educate the public and government officials on the problems and challenges of lockdown and help countries without a tangible plan or standard operating procedures to formulate an effective solution. The New Taipei City Government gained useful experience and learned valuable lessons during the exercise. These experiences will enable government departments to implement lockdown measures in an organized manner, thereby minimizing public panic and ensuring that they are fully prepared before an outbreak occurs. The described tabletop exercise is not designed solely for COVID-19 and can be adapted for other epidemics, such as influenza.
